# Mapping the Rise in Machine Learning in Environmental Chemical Research: A Bibliometric Analysis

**DOI:** 10.3390/toxics13100817

**Published:** 2025-09-26

**Authors:** Bojana Stanic, Nebojsa Andric

**Affiliations:** Department of Biology and Ecology, Faculty of Sciences, University of Novi Sad, Trg Dositeja Obradovica 2, 21000 Novi Sad, Serbia; bojana.stanic@dbe.uns.ac.rs

**Keywords:** machine learning, environmental chemicals, bibliometric analysis, co-occurrence mapping, VOSviewer, human health risk assessment

## Abstract

Machine learning (ML) is reshaping how environmental chemicals are monitored and how their hazards are evaluated for human health. Here, we mapped this landscape by analyzing 3150 peer-reviewed articles (1985–2025) from the Web of Science Core Collection. Co-citation, co-occurrence, and temporal trend analyses in VOSviewer and R reveal an exponential publication surge from 2015, dominated by environmental science journals, with China and the United States leading in output. Eight thematic clusters emerged, centered on ML model development, water quality prediction, quantitative structure–activity applications, and per-/polyfluoroalkyl substances, with XGBoost and random forests as the most cited algorithms. A distinct risk assessment cluster indicates migration of these tools toward dose–response and regulatory applications, yet keyword frequencies show a 4:1 bias toward environmental endpoints over human health endpoints. Emerging topics include climate change, microplastics, and digital soil mapping, while lignin, arsenic, and phthalates appear as fast-growing but understudied chemicals. Our findings expose gaps in chemical coverage and health integration. We recommend expanding the substance portfolio, systematically coupling ML outputs with human health data, adopting explainable artificial intelligence workflows, and fostering international collaboration to translate ML advances into actionable chemical risk assessments.

## 1. Introduction

The assessment of environmental chemicals and their effects on ecosystems and human health has undergone a profound transformation in recent years. Traditional toxicological approaches are increasingly being supplemented or replaced by innovative methodologies aimed at improving efficiency, reducing costs, minimizing animal testing, and enhancing predictive accuracy. Among these advances, the integration of artificial intelligence (AI) has emerged as a particularly powerful development, enabling the analysis of complex, high-dimensional datasets that characterize modern chemical and toxicological research. This evolution reflects a broader shift within toxicology, transitioning from an empirical science focused primarily on apical outcomes to a data-rich discipline ripe for AI integration [1]. AI’s capacity to handle big data facilitates probabilistic predictions and pattern recognition, which are increasingly being applied in chemical risk assessment frameworks [2]. Various AI methodologies have demonstrated significant potential in predicting toxicological endpoints, including effects of chemicals on human receptors [3,4,5,6,7] as well as on critical physiological systems such as steroidogenesis [8], reproduction [9], cardiovascular health [10], or metabolic disorders like diabetes mellitus [11]. Applications of AI are also evolving in environmental monitoring and ecological impact studies [12,13,14,15].

As the use of AI, particularly machine learning (ML), in environmental chemical research accelerates, there is a growing need to systematically characterize how the field is evolving. The literature spans diverse domains, from toxicology and human health to environmental monitoring and fate modeling. At the molecular and cellular level, studies deploy interpretable ML together with classical learners (random forests, support vector machines [SVMs], gradient boosting, k-nearest neighbors [k-NN], and Bayesian models such as Bernoulli naïve Bayes) and deep/multitask neural networks to classify receptor binding, agonism, and antagonism, with large-scale consensus efforts improving robustness and external predictivity [3,4,5,6]. Extending beyond the estrogen receptor, classification models such as k-NN, random forests, and Bernoulli naïve Bayes for the androgen receptor [16] and convolutional neural networks for the progesterone receptor [7] underscore the portability of these approaches to other endocrine targets. At broader environmental scales, ML is widely applied to forecasting water, air, and land quality to support monitoring, early warning systems, and assessments of agricultural and human health impacts. Recent work on graph neural networks (GNNs) that encode river network topology, frameworks for long-term calibration/validation in data-scarce regions, and models such as SVMs, Kolmogorov–Arnold Networks, multilayer perceptrons, and extreme gradient boosting (XGBoost) for drinking water quality index prediction collectively demonstrate breadth and feasibility across spatial scales and data regimes [17,18,19]. For air quality, hybrid directed GNNs with spatiotemporal meteorological fusion, ML-guided integration of fixed and mobile sensors for high-resolution PM2.5 mapping and data-driven modeling of long-range wildfire transport illustrate how modern ML frameworks enhance forecasting and exposure assessment [20,21,22]. For land quality, supervised learners including extremely randomized trees, gradient boosting, XGBoost, SVMs, and tuned random forests augmented with spatial regionalization indices to encode spatial dependence are being used to map heavy-metal contamination from field to global scales, strengthening surveillance and decision-making [23,24]. A bibliometric synthesis is therefore essential to chart how methods and ideas diffuse across these areas, revealing research fronts and cross-disciplinary bridges. By mapping topics, keywords, and collaboration networks, such analyses clarify where evidence is consolidating, where gaps persist, and which datasets and benchmarks anchor the field. This evidence base enables researchers and regulatory agencies to prioritize resources, harmonize standards, and accelerate the translation of ML methods into risk assessment and policy.

Recently, Yarmohammadi et al. employed a bibliometric approach to document the growth of computational toxicology, highlighting a notable increase in publications using the keyword “machine learning” from 2010 onward, with an especially pronounced surge between 2020 and 2024 [25]. In addition to this study, several others have explored computational and AI-based approaches for predicting the toxicity of environmental chemicals. However, these studies are not bibliometric in nature; rather, they offer a more regulatory, conceptual, or future-oriented perspectives on how AI technologies can reshape toxicity prediction. For example, some emphasize the need for integrating AI within regulatory science, calling for standardized datasets, enhanced transparency, and collaborative development of predictive frameworks. Others point to persistent barriers to regulatory acceptance and call for a pragmatic approach in applying quantitative structure–activity relationship (QSAR) models and other computational tools in real-world contexts [26,27].

While our study may appear similar to the above-mentioned works, it differs significantly in scope and methodology. Some studies lack quantitative mapping of AI or ML applications [26,27], while others take a broader perspective that includes a variety of computational tools beyond ML [25]. In contrast, our study offers the first focused bibliometric analysis dedicated specifically to the application of ML in environmental chemical research. By analyzing 3150 publications from the Web of Science Core Collection, we provide a detailed analysis of temporal trends, geographic contributions, research clusters, and emerging thematic areas. Our study introduces several distinct analytical layers that set it apart from previous studies, such as granular thematic mapping of research domains, systematic extraction and co-occurrence analysis of chemical terms, and comparative quantification of environmental versus human health focus. This approach enables a more nuanced understanding of how ML is being employed in environmental and health applications, and it provides evidence-based recommendations for future research and interdisciplinary collaboration. Our analysis complements the studies performed so far in the field of AI and environmental chemicals and may serve as a useful resource in supporting strategic decision-making by funding agencies, research organizations, and policymakers. In the context of rapidly evolving fields, such as the intersection of ML and environmental chemical research, bibliometric studies not only clarify the intellectual landscape but also illuminate pathways for future innovation and collaboration.

## 2. Methods

### 2.1. Dataset Collection

This study employed the Web of Science Core Collection (https://www.webofscience.com/wos/woscc/basic-search, accessed on 16 June 2025) as the primary data source. The search query used was “machine learning” AND “environmental chemicals”. This was applied across all searchable fields. The search was restricted to publications published between 1985 and 2025, limited to article-type documents written in English. The search resulted in a dataset comprising 3150 relevant publications, which served as the basis for all subsequent analyses.

### 2.2. Dataset Analysis

Basic descriptive statistics, such as the annual distribution of publications, author contributions, and institutional affiliations, were generated using the Web of Science built-in data analysis tool. For in-depth bibliometric mapping and network visualization, VOSviewer version 1.6.20 was used. The full records and cited references from the 3150 retrieved articles were imported into VOSviewer to perform several types of analyses, including: (i) co-citation analysis of cited authors, cited sources, and cited references; (ii) co-occurrence analysis of author keywords; and (iii) cluster analysis to identify major thematic structures within the literature. In addition, the R programming environment version 4.2.2 was used for complementary visualizations and statistical analyses. Specifically, R was used to construct temporal keyword evolution maps and identify and visualize the most frequently mentioned and emerging chemicals, based on terms extracted from abstracts, author keywords, and Keywords Plus. This combined approach allowed for both quantitative and network-based insights into the development and structure of the ML domain within environmental chemical research.

## 3. Results and Discussion

### 3.1. Distribution by Year of Publication

**Figure 1:**
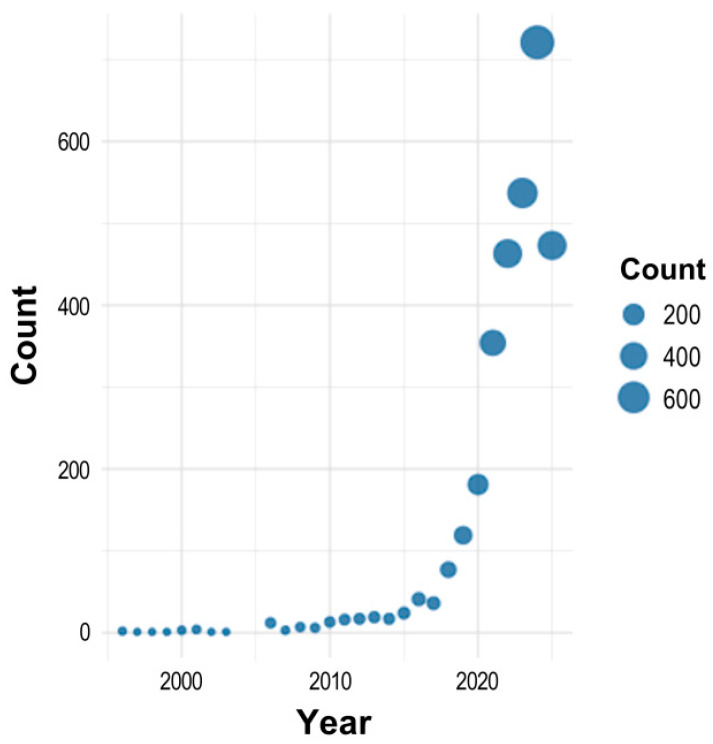
Annual publication trends in ML and environmental chemical research (1996–2025), showing a sharp increase after 2020.

Since 1996, a total of 3150 publications have been recorded in the field of ML and environmental chemical research (Figure 1). Until 2015, annual publication output remained modest, with fewer than 25 papers published per year, indicating limited engagement from research institutions and scientists in this domain. A notable shift occurred in 2020, when the number of publications rose sharply to 179. This upward trend continued, with output nearly doubling in 2021 to 301 publications. Research activity has maintained strong and consistent growth since then, culminating in over 719 publications in 2024. The current output in 2025 (545 publications as of mid-year) suggests that the 2024 record will likely be surpassed, highlighting the field’s accelerating momentum and growing global interest. This publication trend in ML and environmental chemical research domain closely mirrors the trajectory observed in the broader field of computational toxicology characterized by a relatively slow start that has been followed by a marked surge in publication activity over the past two decades [25].

### 3.2. Distribution by Affiliation and Country

**Figure 2:**
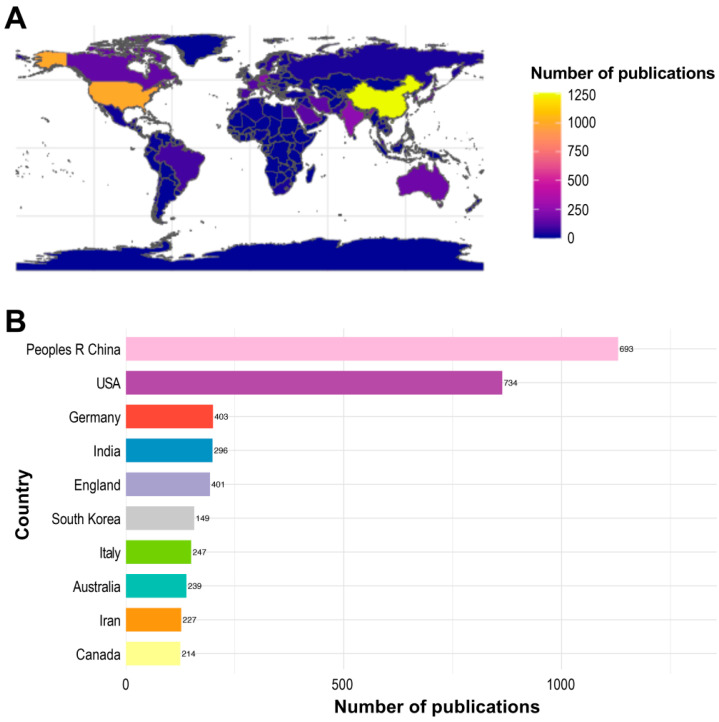
(**A**) Top contributing countries in ML and environmental chemical research based on publication counts (1996–2025). (**B**) TLS values for each country, shown numerically to the right of the corresponding bars.

From 1996 to present, a total of 4254 institutions across 94 countries have contributed to the advancement of ML applications in environmental chemical research. Figure 2 illustrates both the number of publications and total link strength (TLS) for the top 10 contributing countries. Of the 3150 publications identified in this field, the People’s Republic of China leads with 1130 publications and a TLS of 693, indicating its dominant role in shaping this research area. The United States ranks second, with 863 publications and a higher TLS of 734, suggesting a stronger collaborative network. Other prominent contributors include India (255 publications), Germany (232 publications), and England (229 publications) (Figure 2A,B). The remaining countries presented in Figure 2B each account for a smaller proportion of the total research output yet collectively underscore the field’s growing global engagement.

Table 1 presents the top 10 affiliations ranked by publication output in the field of ML applications in environmental chemical research. The Chinese Academy of Sciences leads with 174 publications over the past decade, followed by the United States Department of Energy with 113 publications. Major contributions have also come from other prominent institutions in the United States and China, Switzerland, and France, including the University of California System (106 publications), the University of Chinese Academy of Sciences (75 publications), the Swiss Federal Institutes of Technology Domain and Tsinghua University (64 publications), and the French Centre National de la Recherche Scientifique with 57 publications.

Table 2 shows the top 10 authors by the number of publications in the field of ML and environmental chemical research. All identified authors are affiliated with institutions in China or the United States, with six authors based in China (Dalian University of Technology; East China University of Science and Technology—three authors; Nankai University; Yangzhou University) and four in the United States (Tulane University; Emory University; Case Western Reserve University; United States Food and Drug Administration). A cohesive China-centered node is evident at East China University of Science and Technology (Weihua Li, Guixia Liu, Yun Tang; each with 13 publications), while the United States node spans academic and regulatory affiliations, suggesting a translational pipeline from method development to regulatory application. This geography mirrors the institutional concentration reported in Table 1 and likely reflects access to curated datasets, AI infrastructure, and sustained funding in these two countries. In terms of productivity, Jingwen Chen (Dalian University of Technology) leads with 15 papers. When network centrality is considered (TLS), the field is anchored by a China–United States axis: Hao Zhu from Tulane University and Weihua Li, Guixia Liu, and Yun Tang from East China University of Science and Technology are highly connected. On citation impact, Huixiao Hong (United States Food and Drug Administration) ranks highest (771 citations) despite fewer publications and a modest TLS (499), indicating influential outputs that are less embedded in the co-authorship network. Yun Tang (676 citations) likewise stands out for impact relative to his TLS, whereas Huichun Zhang exhibits a bridge profile (10 papers, high TLS = 1531), suggesting cross-cluster connectivity.

### 3.3. Cooperation Network Relationship Analysis

**Figure 3:**
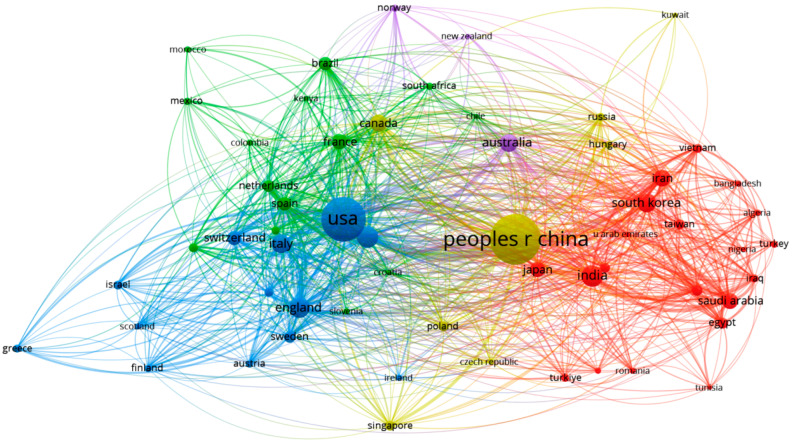
Co-authorship network of countries in ML and environmental chemical research, with clusters representing collaborative relationships (minimum 10 publications per country).

**Figure 4:**
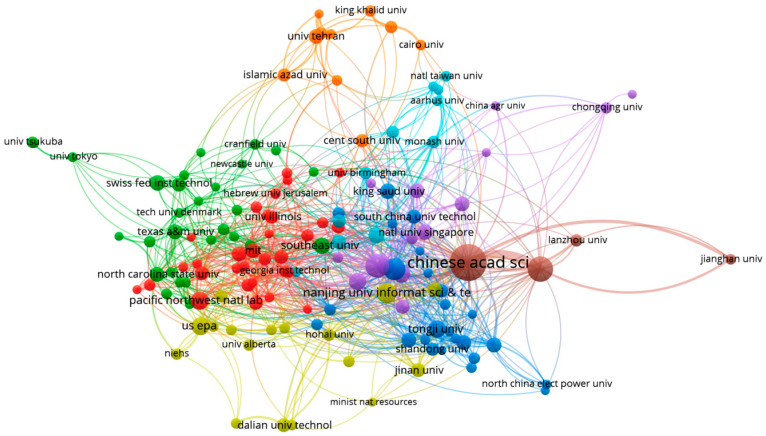
Institutional co-authorship network in ML and environmental chemical research, showing major collaboration clusters (minimum 10 documents per institution).

To elucidate the collaborative dynamics among countries and institutions, a co-authorship analysis was conducted using VOSviewer, resulting in superimposed visual network maps presented in Figure 3 and Figure 4. In these networks, nodes (depicted as circles) represent countries (Figure 3) or affiliations (Figure 4), while clusters are distinguished by different colors. The size of each circle reflects the publication output of a country or institution, and the connecting lines represent collaborative links.

Figure 3 illustrates the co-authorship network among countries/regions, based on a minimum threshold of 10 documents per country. A total of 58 countries met this criterion, forming 872 collaborative links with a combined TLS of 3637. Of note, countries such as the People’s Republic of China, the United States, India, Germany, and England exhibit strong international collaboration within this research domain. The network reveals that while ML-related research on environmental chemicals is globally distributed, certain countries demonstrate more frequent and cohesive collaborations, forming distinct clusters. For example, China, a leading contributor, frequently collaborates with Canada, the Czech Republic, Hungary, and Singapore. Together, these countries form Cluster 4. In contrast, the United States tends to collaborate more closely with Germany, England, Italy, and others, forming Cluster 3. Comparative analysis of these clusters shows that Cluster 4 (led by China) comprises 1130 publications and a TLS of 693, while Cluster 3 (led by the United States) has a slightly higher TLS of 734 but a lower number of documents—864. These differences highlight not only the productivity but also the varying collaborative strategies among leading nations in this field. For better cluster differentiation, see Supplementary Figure S1.

In the co-authorship analysis of institutional collaboration, a threshold of at least 10 publications per organization was applied, resulting in 142 organizations meeting this criterion. The Chinese Academy of Sciences emerged as the most prolific contributor, with 64 collaborative links. Further analysis of the network structure reveals the presence of distinct clusters based on collaboration strength. One prominent cluster revolves around the Chinese Academy of Sciences and includes other Chinese institutions such as Jianghan University, the University of Chinese Academy of Sciences, and Lanzhou University, reflecting strong intra-national collaboration. Additionally, another predominantly Chinese cluster comprises several other universities and research organizations within China. While these clusters exhibit strong internal collaboration, their connections with institutions from Europe and the United States appear comparatively weaker. In contrast, two other major clusters are primarily composed of institutions from the United States and Europe, including MIT, the University of Illinois, Georgia Tech, the United States Environmental Protection Agency (US EPA), Hebrew University, and the Swiss Federal Institute of Technology. These institutions not only maintain strong regional ties but also demonstrate active collaboration with organizations from other regions globally (Figure 4). For better cluster differentiation, see Supplementary Figure S2.

### 3.4. Cited Sources Co-Citation Analysis

**Figure 5:**
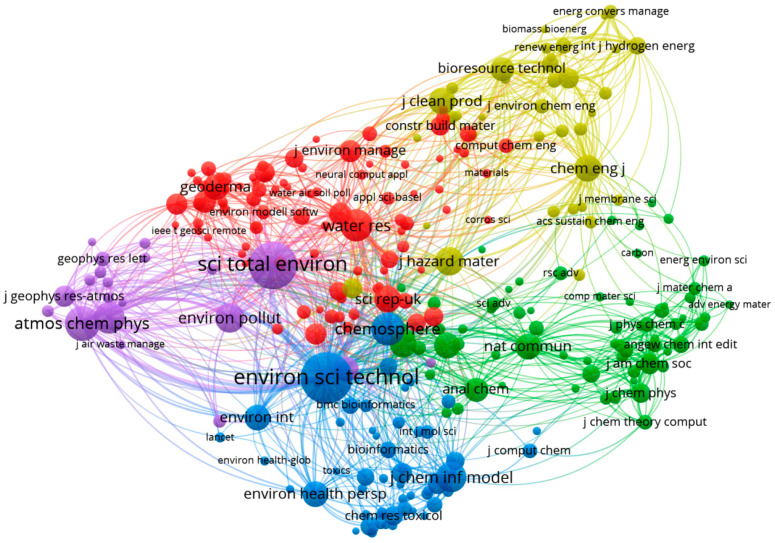
Co-citation network of cited sources (journals) with more than 100 citations.

Figure 5 presents a co-citation analysis map of cited sources, generated using VOSviewer. For this analysis, sources that were cited more than 100 times were selected, resulting in a total of 276 sources out of 28,769 meeting this threshold. In the visualization, each node represents a cited source, while the links between nodes indicate co-citation relationships, i.e., instances where two sources were cited together in the same document. This mapping highlights the intellectual structure of the field by identifying influential sources and the strength of their interconnectedness. For better cluster differentiation, see Supplementary Figure S3.

Table 3 highlights the most influential journals, distributed across five distinct clusters as shown in Figure 5, based on co-citation analysis. Only journals with more than 100 citations were included. These journals generally possess high impact factors, with most classified in Q1 Category Quartile, underscoring their prominence in the field. *Environmental Science and Technology* ranks first in terms of TLS, followed by *Science of the Total Environment*, *Atmospheric Chemistry and Physics*, and others. All top 10 journals, ranked by TLS, have relatively high impact factors, with *Chemical Engineering Journal* reporting the highest (13.2), while *Atmospheric Environment* has the lowest impact factor among them (3.7). Geographically, the majority of these leading journals are based in Europe, particularly in the Netherlands, which hosts several of them.

**Figure 6:**
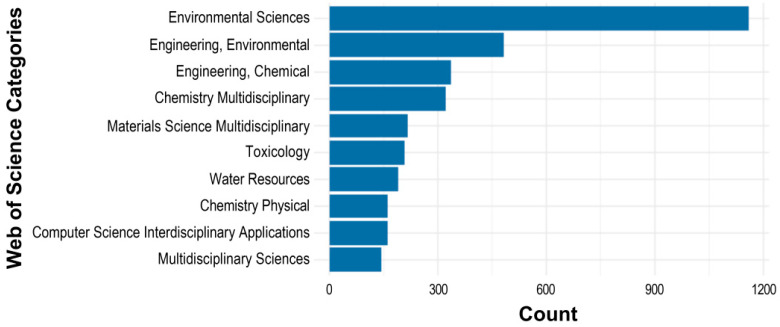
Top 10 Web of Science categories contributing to ML and environmental chemical research, ranked by number of publications.

Thematically, the journals shown in Table 3 primarily focus on the environmental sciences, with many also intersecting with engineering and public health disciplines. This finding aligns with the Web of Science category rankings (Figure 6), in which *Environmental Sciences* holds the top position, followed by *Engineering*, *Environmental* and *Engineering*, *Chemical*. Additionally, *Multidisciplinary Sciences* is also represented among the top 10 categories contributing to the ML and environmental chemicals research domain. These results are consistent with the scope of the *Environmental Sciences* category, which emphasizes research on complex environmental challenges, including those related to human health. Many of the top-ranked journals in this field publish innovative studies using novel approaches and cutting-edge technologies for assessing chemical toxicity more effectively and efficiently.

### 3.5. Cited References Co-Citation Analysis

**Figure 7:**
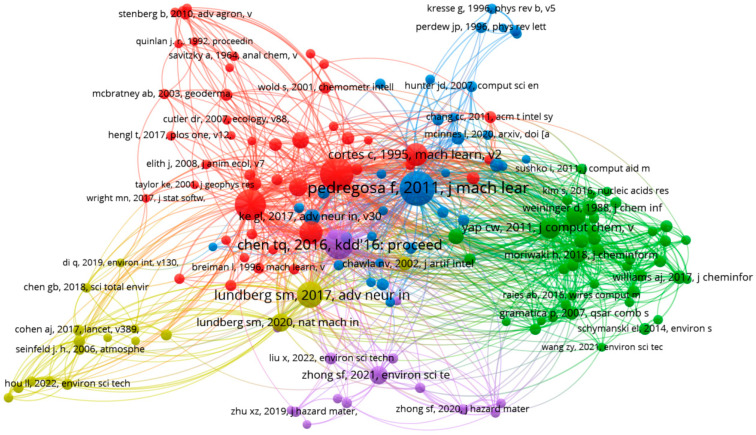
Co-citation network of the 154 most cited references (minimum 20 citations) in ML and environmental chemical research, with clustering based on citation links.

Figure 7 presents the co-citation analysis of cited references, conducted using VOSviewer software. This analysis explores the relationships among specific references frequently cited together in the literature. In the resulting network map, each node represents a cited reference, and the links between nodes indicate co-citation relationships. Nodes sharing the same color belong to the same cluster, signifying closely related references. For this study, references cited more than 20 times were included in the analysis. Out of 153,738 references in the dataset, 154 met the selection criteria. The cluster map provides a visual representation of the spatial distribution and grouping of these references (Figure 7). For better cluster differentiation, see Supplementary Figure S4.

Table 4 lists the top 10 most highly cited references during the study period, ranked by TLS. These references form the conceptual foundation for the development and application of ML, spanning from fundamental algorithms and tools to domain-specific applications in chemistry, ecology, and medicine. Several highly cited papers introduce foundational ML algorithms and methodologies, such as random forest [28], support SVMs [29], and explainable AI for tree-based models [30]. In addition, significant attention is devoted to software tools and libraries, such as Python, caret, PaDEL-descriptor, and molecular fingerprints, which facilitate the implementation of ML approaches in chemical and biological research. Of note, some of the most influential works address domain-specific applications, including methods for molecular structure encoding [31], the use of kernel tricks in SVMs for high-dimensional data classification [29], and toolkits for computing molecular descriptors widely used in QSAR modeling [32]. These core methodologies and software frameworks have been instrumental in advancing the application of ML in assessing the effects of chemicals on human and environmental health, thus forming a critical backbone of the field.

### 3.6. Research Direction Analysis

**Figure 8:**
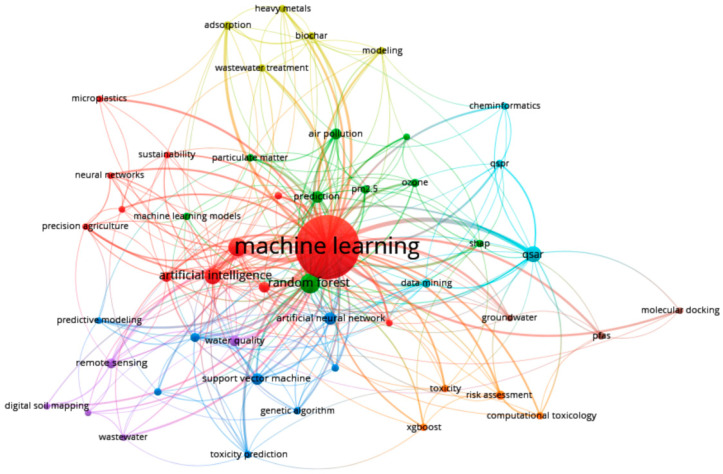
Keyword co-occurrence network for the top 50 terms in ML and environmental chemical research, with clusters representing distinct thematic domains.

A co-occurrence analysis of keywords related to ML applications in environmental chemicals research was performed in VOSviewer. The analysis parameters were set to select 50 keywords that met the inclusion criteria. Clustering was conducted by VOSviewer, resulting in the co-occurrence network graph shown in Figure 8. The analysis revealed that the selected keywords were grouped into eight distinct clusters.

Based on the clustering results presented in Figure 8, the thematic research directions and corresponding keyword groups are summarized in Table 5. The VOSviewer algorithm identified eight keyword clusters. Leading keywords within each cluster were determined using Term Frequency-Inverse Document Frequency (TF-IDF) values calculated in R. In the first three clusters (Clusters 1, 2, and 3), the leading keywords—*machine learning*, *deep learning*, and *random forest*, respectively—reflect core AI tools. In contrast, the remaining five smaller clusters (Clusters 4–8) are centered around domain-specific applications, with leading keywords being *adsorption*, *water quality*, *QSAR*, *risk assessment*, and *PFAS*. This distribution suggests that these clusters primarily represent the practical implementation of AI methodologies within specific environmental contexts. However, the presence of domain-specific keywords as cluster leaders does not imply the absence of AI tools within those clusters. For instance, in Cluster 8, although *PFAS* emerged as the leading keyword, AI methods played a central role in the research. Multiple ML models were employed to assess the predictive value of key PFAS compounds, namely perfluorooctane sulfonate and perfluorooctanoic acid, in relation to metabolic syndrome [33]. Similarly, *risk assessment*, the leading term in Cluster 7, is examined in the context of AI-driven methodologies [34], further emphasizing the integrative role of ML across these thematic domains.

To further explore the coherence of the identified clusters, we conducted a Silhouette analysis to assess how well each keyword aligns with its assigned cluster compared to others. The results demonstrated that the average distance of each keyword to its own cluster was substantially lower than its distance to other clusters, indicating strong within-cluster similarity. This suggests that the research directions represented by the eight clusters are well-defined and largely distinct, with minimal thematic overlap between them.

### 3.7. Evolution of Research Directions

**Figure 9:**
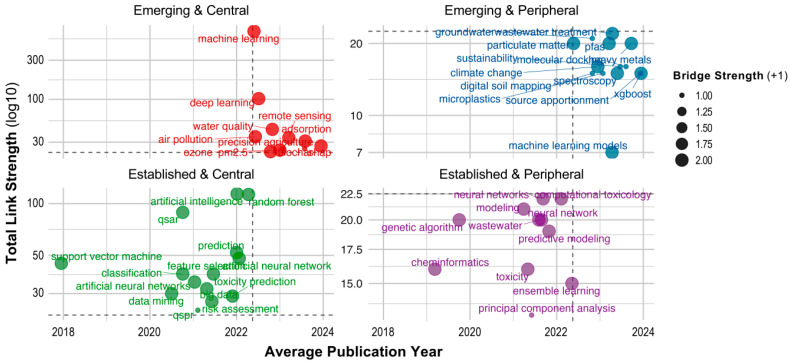
Quadrant analysis of the top 50 keywords based on total link strength (TLS) and novelty (pre/post 2022), identifying emerging and established research themes.

To better understand the thematic structure and dynamics of research in the intersection of ML and environmental chemical research, we analyzed the positions of the top 50 keywords within the co-occurrence network (Figure 9). The visualization is organized into four quadrants based on two criteria: term novelty (before or after 2022) and TLS, which is a measure of a keyword’s centrality in the network. The quadrants are defined as follows: (i) Emerging and Central (upper right)—recently introduced keywords (>2022) that already hold strong connectivity within the network (TLS > 20); (ii) Emerging and Peripheral (lower right)—new keywords (>2022) that are still developing their position (TLS < 20); (iii) Established and Central (upper left)—older terms (<2022) that remain core elements of the field (TLS > 20); and (iv) Established and Peripheral (lower left)—older keywords (<2022) that used to be in focus earlier but are becoming less prominent now (TLS < 20). The analysis reveals several Emerging and Central keywords such as *machine learning*, *deep learning*, *remote sensing*, *air pollution*, and *water quality*, indicating that these “hot topics” are currently at the forefront of research. Keywords like *climate change*, *particulate matter*, *groundwater*, *digital soil mapping*, *wastewater*, and *PFAS* fall into the Emerging and Peripheral category. These are novel but still peripheral topics, showing potential for future growth and integration into the core of the research field. In contrast, established terms such as *artificial intelligence*, *random forest*, *QSAR*, *predictive modeling*, and *support vector machine* remain central and foundational to the domain. Meanwhile, keywords like *genetic algorithm*, *toxicity*, *principal component analysis*, and *predictive modeling* appear in the Established and Peripheral quadrant, suggesting a gradual decline in their prevalence. This quadrant-based analysis indicates a shift in research focus within the ML-environmental chemicals domain. While ML models and AI methodologies remain crucial, the application domains are changing; there is growing interest in areas such as water analysis, climate change, soil contamination, and microplastics. In particular, *water analysis* appears in both emerging quadrants, highlighting its continued relevance and expanding application. Keywords like *climate change*, *digital soil mapping*, and *microplastics* are present exclusively in the Emerging and Peripheral quadrant, indicating new but promising directions in the field. Since 2022, the term *climate change* has appeared in 24 publications, including studies that explore its linkage with environmental ML modeling [35,36]. The keyword *microplastics* has been used in 3 publications to date. Given the rising environmental and human health concerns due to microplastic pollution and exposure [37], ML-based models are increasingly applied to predict and assess risks posed by microplastics [38,39]. Similarly, *digital soil mapping*, which has appeared in 13 publications to date, is becoming more prominent as ML techniques are increasingly applied to assess the content and distribution of potentially toxic elements in soil impacted by anthropogenic activities [40,41,42]. In summary, this quadrant analysis provides a strategic perspective on the emerging and shifting research priorities, highlighting how ML applications in environmental science are evolving over time.

**Figure 10:**
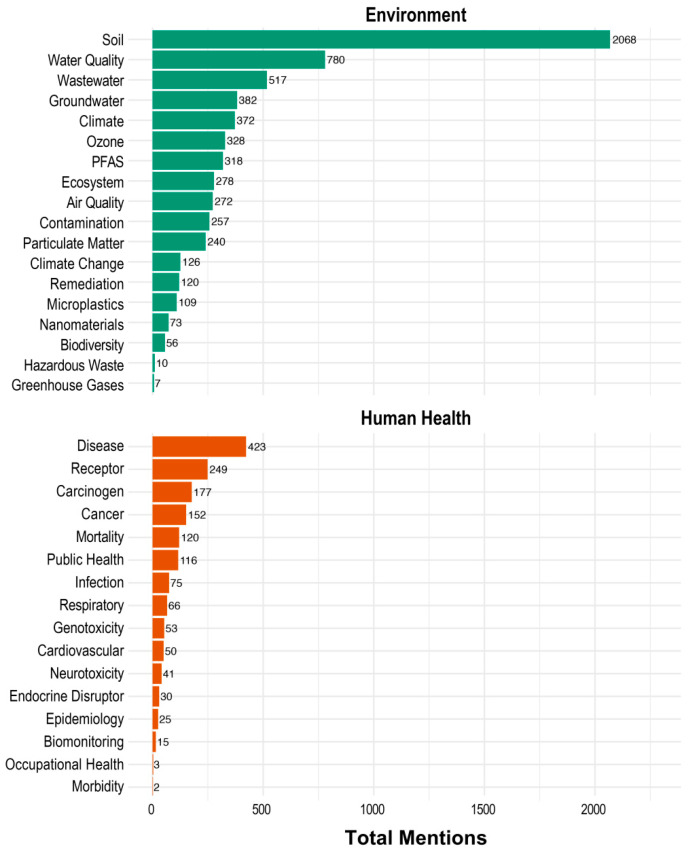
Frequencies of environmental and human health-related terms in abstracts, keywords, and keywords plus, illustrating thematic focus in 3150 publications.

To further explore how ML is applied in the context of environmental versus human health research, we analyzed the content of 3150 documents by screening their abstracts, author keywords, and Keywords Plus using a predefined set of terms (the full list is shown along the y-axis of Figure 10). These terms were selected to represent key thematic elements of environmental and human health research. The analysis yielded a total of 6313 hits for environmental terms and 1597 hits for human health terms, indicating a greater emphasis on environmental applications of ML within the literature. Within each thematic group, the frequency of individual terms varied markedly. In the environmental domain, *soil* was by far the most frequently mentioned term, with 2068 hits, followed by *water quality* (780 hits). In contrast, terms such as *greenhouse gases*, *hazardous waste*, and *biodiversity* were mentioned in fewer than 100 documents. Similarly, within the human health domain, *diseases* had the highest frequency (423 hits), followed by *receptor* (249 hits). However, terms like *morbidity*, *occupational health*, and *biomonitoring* appeared much less frequently, each with fewer than 20 mentions (Figure 10). These findings highlight that the use of ML methodologies in environmental chemical research is more frequently focused on environmental endpoints than on human health outcomes. Moreover, the dominance of terms like *soil* and *water quality* aligns with the thematic trends identified in Figure 9. It is important to note that this analysis is limited by the predefined list of search terms. While commonly used keywords such as *soil*, *water quality*, *diseases*, and *receptors* were included, the omission of additional relevant terms may have led to an underrepresentation of certain aspects, which may have affected the relationship between environmental and human health themes. Therefore, while the findings suggest a stronger orientation toward environmental applications of ML, they should be interpreted with caution, bearing in mind this methodological limitation.

### 3.8. Translational Implications for Human Health Risk Assessment

Although our term frequency analysis has shown a striking 4:1 imbalance between environmental and human health research focus (6313 environment-related keyword hits versus 1597 human health hits), the keyword co-occurrence map nonetheless reveals a discrete Risk Assessment cluster (Cluster 7) built around terms such as *risk assessment*, *computational toxicology*, *toxicity*, and *XGBoost* (Table 5). The presence of this cluster indicates that tools originally developed for environmental monitoring are now migrating toward endpoints directly relevant to human health risk assessment. The literature already offers proof-of-concept models that predict receptor binding and pathway level toxicity (e.g., estrogen and progesterone receptors, steroidogenesis, cardiotoxicity, diabetes risk) with encouraging accuracy and mechanistic insight [3,4,5,6,7,8,9,10,11].

Building on these advances, several studies have illustrated how environmental ML innovations actively accelerate human health risk assessment. Mixture-learning models link multi-chemical exposures to metabolic syndrome outcomes in adults [33], while a pathophysiology-based New Approach Method provides dose–response points of departure for complex contaminant mixtures [43]. High-throughput classifiers accurately predict hematotoxicity [44] and receptor-mediated endocrine disruption, such as PFAS binding to peroxisome proliferator-activated receptor alpha [45]. The routine use of SHAP and other explainable AI techniques [30] further enhances transparency, allowing model outputs to be interrogated by regulators. Collectively, these advances outline an end-to-end pipeline in which: (i) large chemical inventories are screened in silico for hazard; (ii) pathway-anchored models translate those hazards into dose–response metrics; and (iii) explainable AI frameworks communicate model uncertainty, thereby accelerating human health risk assessment.

### 3.9. Emerging Environmental Chemicals

**Figure 11:**
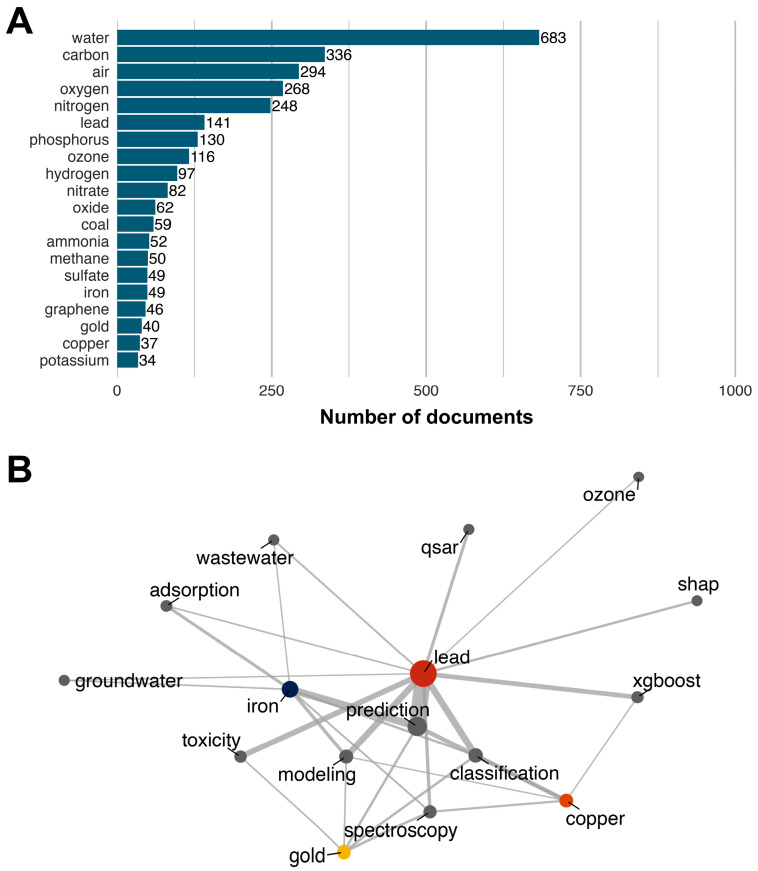
(**A**) Top 20 most frequently mentioned chemical terms. (**B**) Co-occurrence network between four selected heavy metals (lead, iron, copper, and gold) and the top 50 thematic keywords.

To identify the most frequently studied chemical terms within the domain of ML and environmental chemical research, we utilized the DSSTox_Synonyms dataset from the EPA-ORD-CCTE Clowder repository (https://clowder.edap-cluster.com, accessed on 30 June 2025), a comprehensive and scalable resource containing over one million chemical names and synonyms. This chemical list was used to screen the abstracts, author keywords, and Keywords Plus of the 3150 documents retrieved from the Web of Science using a document frequency approach. From this process, 551 unique chemical terms were identified. Of these, 315 terms appeared in only a single document, 194 terms appeared between 2 and 20 times, and 42 terms were found in more than 20 documents. This distribution suggests that ML applications in environmental chemical research are focused on a relatively small subset of chemicals. The top 20 most frequently mentioned chemical terms are presented in Figure 11A. *Water* ranked highest with 683 mentions, followed by *carbon* (336 mentions) and *air* (294 mentions). While *water* and *air* are environmental terms rather than discrete chemicals, and *carbon* may appear as part of molecular structures rather than as an independent chemical of study [45], the list also includes some of the prominent heavy metals such as *lead*, *iron*, and *copper*. This is in line with the previously observed focus on environmental themes and topics such as water and air quality in recent ML-driven research.

To further examine the relationship between frequently mentioned chemicals and thematic topics, we constructed a co-occurrence network (Figure 11B) linking four selected heavy metals from the top 20 list, namely *lead*, *iron*, *copper*, and *gold*, with the top 50 keywords identified in the co-occurrence network presented in Figure 9. Other top-ranked terms such as *carbon* or *phosphorus* were excluded from this analysis due to their ambiguous contextual usage (e.g., structural references rather than chemical investigation). The results show differences in connectivity among the four selected heavy metals. *Lead* and *iron* exhibited multiple strong associations (link strength > 5) with the thematic keywords, whereas *copper* and *gold* showed more limited linkages. For example, *iron* was prominently linked with *groundwater*, and *lead* showed strong connections with themes such as *ozone* and *prediction*, indicating its use in predictive modeling studies. Of note, *lead* was associated with both environmental and toxicological/health-related research, including studies on wastewater pollution [46], prediction of chemical mixture toxicity and carcinogenicity [43], mitochondrial activity [47], and QSAR modeling [48]. In contrast, *copper* exhibited fewer and more focused associations, predominantly within environmental research themes. Examples include *soil analysis within the classification* theme [49], *ecotoxicological effects of copper oxide nanomaterials* within the *modeling* theme [50], and *soil heavy metal concentration* in the *spectroscopy* theme [51]. This analysis illustrates that while some terms, such as *lead* and *iron*, are broadly integrated across multiple research themes, others like *copper* and *gold* are more narrowly applied, particularly in environmental monitoring and modeling. We would like to emphasize that this analysis was limited to co-occurrences between selected chemical terms from the top 20 list and the top 50 thematic keywords. Interactions with other potentially relevant terms in the abstracts or keyword sets were not considered. Additionally, the co-occurrence results are sensitive to the thresholds used for filtering, such as the minimum number of documents in which a co-occurrence must appear. Adjusting these thresholds could yield different patterns of association and deserves exploration in future analyses.

### 3.10. Evolution of Keywords

**Figure 12:**
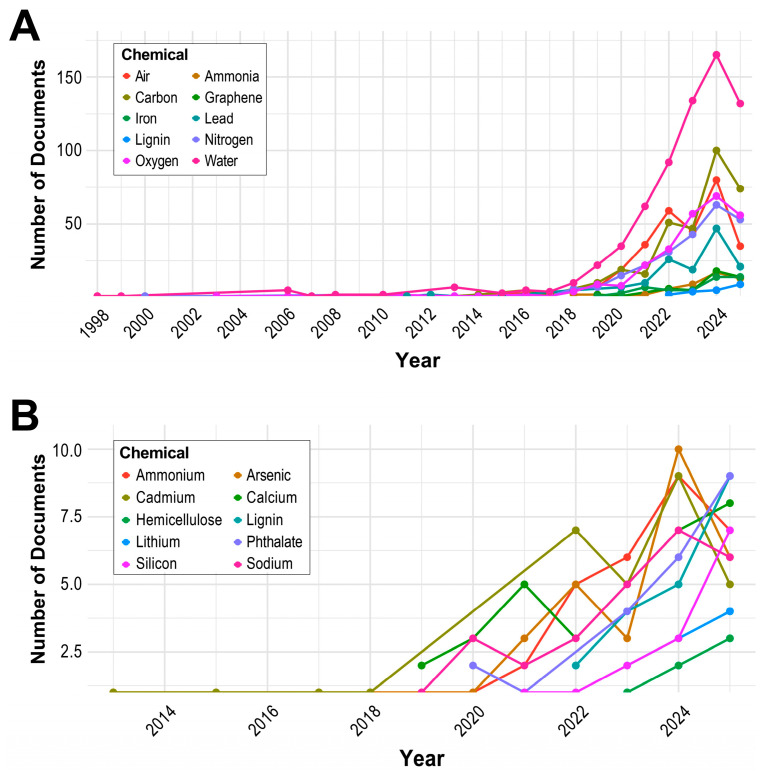
Frequencies of emerging chemical terms across years within the domain of ML and environmental chemical research. (**A**) Yearly trends of the top 10 emerging chemical terms based on regression slope and *p*-value. (**B**) Yearly trends of the emerging chemicals beyond the top 20.

To identify emerging chemical terms in the domain of ML and environmental chemical research, we analyzed term frequencies across years using document frequency from abstracts, author keywords, and keywords plus. Terms occurring fewer than five times in at least three separate years were excluded from the analysis. From the remaining terms, we selected the top 20 emerging chemicals based on the slope and *p*-value obtained from simple linear regression analysis of yearly frequency trends. Figure 12A shows a rise in research activity for these ten terms starting in 2018, with a sharp increase after 2020. Interestingly, nine out of the ten emerging terms were also among the top 20 most frequently mentioned chemical terms, indicating not only their cumulative frequency but also their accelerating presence in recent years. One exception is *lignin*, which does not appear among the top 20 frequent terms but demonstrates a consistent increase in occurrence beginning in 2022. This term is often associated with biomass and bio-oil analysis, suggesting that biomass research is emerging as a relevant theme [52,53].

To uncover additional emerging chemical terms beyond those dominating the top 20, we repeated the same linear regression approach excluding the top 20 chemicals. This allowed us to detect chemicals with more recent and potentially specialized emergence. As shown in Figure 12B, *arsenic* displayed a marked increase in 2023 and 2024, corresponding to its well-known status as a widespread water contaminant (https://www.who.int/news-room/fact-sheets/detail/arsenic, accessed on 3 July 2025) and the ongoing application of ML in water quality assessment [12,54]. Its 2025 frequency appears lower than in 2024; however, because our corpus extends only to mid-2025, it remains unclear whether interest will continue to rise over the remainder of the year or will it plateau. In contrast, *phthalates* show a steady increase from 2021 onward, and their mid-2025 frequency already exceeds the total for 2024, indicating growing prominence in both environmental and human health research. While some documents explore phthalate exposure in environmental contexts [55,56,57], the majority of studies apply ML techniques to investigate health-related outcomes, including risks of hyperuricemia [58], attention deficit hyperactivity disorder [59], neurodevelopment [60], and chronic kidney disease [61]. Furthermore, phthalates were studied in the context of chemical mixtures affecting diabetes [62] and metabolic syndrome [63].

While the identification of emerging chemical terms provides valuable insights into shifting research priorities, this analysis is subject to important limitations. Specifically, chemical names that are not explicitly mentioned in abstracts, author keywords, or Keywords Plus were not captured by our frequency-based extraction method. For instance, several studies analyzing the effect of environmental chemicals on estrogen receptor [3,4,5] and progesterone receptor [7] without naming specific compounds. Consequently, these relevant chemicals are inadvertently excluded, which may lead to underrepresentation of their roles in ML-based research. This reflects a common limitation of bibliometric analyses, which rely on the visibility of terms in metadata and abstracts. In conclusion, our analysis reveals both well-established and emerging chemicals in the ML and environmental chemical research domain. However, the use of broader data extraction strategies, such as full-text mining, could enhance the depth and accuracy of such assessments in future studies.

## 4. Conclusions

This bibliometric map of 3150 Web of Science records documents the accelerating use of ML across environmental chemical research and its early migration toward human health risk assessment. Our findings reveal a rapid increase in research output since 2020, with China and the United States leading in both the number of publications and international collaboration. The majority of publications are concentrated in high-impact environmental science journals. Eight thematic clusters dominate the field, including ML model development, water quality prediction, QSAR applications and PFAS, while a newly formed risk assessment cluster points to emerging dose–response and regulatory applications. Keyword frequencies, however, reveal a 4:1 bias toward environmental endpoints versus human health endpoints, and several high-concern contaminants, such as lignin, arsenic, and phthalates, remain underexplored. When considering these insights, two limitations should be taken into account. First, the reliance on title-abstract keywords may undercount studies that employ ML but do not label methods explicitly. Second, health-related terms are less standardized than ecological descriptors, potentially masking additional studies pertaining to human health. Addressing these gaps will require large-scale, interdisciplinary collaboration that can harmonize chemical and toxicological ontologies, integrate exposure and biomonitoring datasets with ML-ready health outcomes, and embed explainable AI workflows to satisfy regulatory transparency. Expanding chemical coverage, particularly to mixture scenarios and non-ubiquitous yet high-risk compounds, will further align ML innovation with public health protection. Such efforts will ensure more comprehensive and impactful applications of ML in environmental chemical research.

To strengthen feasibility, we anchor our recommendations in concrete EU regulatory use cases. Under the Registration, Evaluation, Authorisation and Restriction of Chemicals (REACH), the European Chemicals Agency’s Guidance on Information Requirements and Chemical Safety Assessment, Chapter R.6 (QSARs and grouping of chemicals) already defines acceptance criteria—defined endpoint, transparent algorithm, applicability domain, performance, and mechanistic interpretation, where possible—so validated ML-based QSARs can be piloted within existing acceptance rules [64,65,66]. Under the Drinking Water Directive (EU) 2020/2184, Member States must set and monitor group parameters for PFAS by 12 January 2026. ML can combine liquid chromatography–high-resolution mass spectrometry non-target data with historical series to prioritize monitoring sites and forecast exceedance risk for targeted sampling and treatment upgrades [67]. The recast Urban Wastewater Treatment Directive (EU) 2024/3019 tightens requirements for monitoring and advanced treatment and introduces new obligations relevant to micropollutants [68]. Here, ML supports optimization of advanced/“fourth-stage” treatment (e.g., ozonation, granular activated carbon) and anomaly detection in plant operations. The microplastics restriction (Commission Regulation (EU) 2023/2055) enables ML-assisted product classification, hotspot detection in influent/effluent, and compliance tracking across supply chains [69]. Finally, the EU AI Act (Regulation (EU) 2024/1689) provides a governance layer for high-impact ML tools (data management, transparency, risk management [70].

Beyond the EU, analogous regulatory pathways exist that make our recommendations feasible internationally. In the United States, EPA’s final PFAS National Primary Drinking Water Regulation sets enforceable maximum contaminant levels for several PFASs and their mixtures. ML can prioritize monitoring, forecast exceedances, and optimize treatment responses using data streams from the fifth Unregulated Contaminant Monitoring Rule (2023–2025) [71,72,73]. In parallel, EPA’s New Approach Methods (NAMs) Work Plan provides a policy pathway for integrating QSAR/read-across and other non-animal methods into chemical decisions, aligning with the Organisation for Economic Co-operation and Development’s validation principles [74]. In the United Kingdom, UK REACH explicitly permits Annex 11 adaptations (e.g., read-across, QSAR) and Health and Safety Executive guidance encourages minimizing vertebrate testing, thereby providing a clear acceptance route for validated ML-based predictions [75,76]. Under Canada’s Canadian Environmental Protection Act/Chemicals Management Plan, Health Canada and Environment and Climate Change Canada formally employ analog/read-across approaches in substance assessments, which can be supported by ML-assisted similarity and endpoint prediction [77,78]. Australia’s Australian Industrial Chemicals Introduction Scheme similarly recognizes read-across/grouping to fill data gaps for hazard characterization, which is another integration point for ML-based QSAR and category inference [79,80]. Finally, under Japan’s Chemical Substance Control Law, the regulators (Ministry of Economy, Trade and Industry; Ministry of Health, Labor and Welfare; Ministry of the Environment) maintain QSAR resources and guidance for bioconcentration/biodegradation, enabling ML-supported predictions to complement testing of new/existing chemicals [81,82]. These examples show that the regulatory “hooks” for ML are already present across multiple jurisdictions. Concrete entry points (screening, read-across support, exceedance forecasting, treatment optimization, and anomaly detection) are therefore immediately actionable with appropriate validation and documentation.

In conclusion, ML has already transformed how scientists characterize environmental chemicals. The next step is to couple those advances with robust human health datasets and transparent algorithms. Doing so will create an end-to-end pipeline that screens large chemical inventories in silico, converts hazards into dose-response metrics, and communicates uncertainty clearly to regulators, thereby accelerating ML uptake where it matters most to public health.

## Figures and Tables

**Table 1:** Top 10 institutions contributing to ML and environmental chemical research (1996–2025), ranked by number of publications and percentage of total output.

Institution	Number of Publications	% of Total Output
Chinese Academy of Sciences	174	5.55
United States Department of Energy	113	3.62
University of California System	106	3.39
University of Chinese Academy of Sciences	75	2.41
Swiss Federal Institutes of Technology Domain	64	2.03
Tsinghua University	64	2.03
Centre National De La Recherche Scientifique	57	1.81
Zhejiang University	55	1.75
Helmholtz Association	50	1.58
Egyptian Knowledge Bank	49	1.56

**Table 2:** Top 10 authors contributing to ML and environmental chemical research (1996–2025), ranked by number of publications.

Author Name	Affiliation	Number of Publications	Citations	TLS
Jingwen Chen	Dalian University of Technology, Dalian, China	15	215	1923
Hao Zhu	Tulane University, New Orleans, United States	13	512	3441
Weihua Li	East China University of Science and Technology, Shanghai, China	13	512	3441
Guixia Liu	East China University of Science and Technology, Shanghai, China	13	512	3441
Yun Tang	East China University of Science and Technology, Shanghai, China	13	676	1156
Yang Liu	Emory University, Atlanta, United States	12	184	221
Yinchang Feng	Nankai University, Tianjin, China	11	263	368
Huichun Zhang	Case Western Reserve University, Cleveland, United States	10	277	1531
Huixiao Hong	US Food and Drug Administration, Jefferson, United States	10	771	499
Tengyi Zhu	Yangzhou University, Yangzhou, China	10	52	379

**Table 3:** Top 10 journals based on co-citation analysis, including TLS, citations, impact factors, and category quartile.

Journal	Country	Cluster	Links	TLS	Citations	Impact Factor	Category Quartile
*Environmental Science and Technology*	USA	3	275	190,071	4665	11.3	Q1
*Science of the Total Environment* ^a^	Netherlands	5	275	177,342	3959	8.0	Q1
*Atmospheric Chemistry and Physics*	Germany	5	229	93,894	2156	5.1	Q1
*Chemosphere* ^b^	Netherlands	3	275	92,746	1931	8.1 ^c^	Q1
*Atmospheric Environment*	UK	5	264	72,877	1701	3.7	Q1
*Environmental Pollution*	Netherlands	5	272	71,492	1514	7.3	Q1
*Water Research*	Netherlands	1	273	70,609	1795	12.4	Q1
*Journal of Hazardous Materials*	Netherlands	4	275	67,983	1405	11.3	Q1
*Chemical Engineering Journal*	Switzerland	4	273	67,687	1245	13.2	Q1
*Journal of Cleaner Production*	Netherlands	4	273	60,527	1233	10.0	Q1

^a^—‘On Hold’ status at the point of Journal Citation Report (JCR) release in June 2025. ^b^—Delisted from the Web of Science Master Journal List on 16 December 2024. ^c^—JCR year 2023.

**Table 4:** Top 10 most highly cited references in the dataset (1996–2025), ranked by TLS, with citation counts and clustering information.

Name	Cluster	Links	TLS	Citations
Pedregosa, F., Varoquaux, G., Gramfort, A., Michel, V., Thirion, B. et al. 2011. Scikit-learn: Machine Learning in Python. *Journal of Machine Learning Research* 12(85), 2825–2830.	3	141	935	303
Breiman, L. 2001. Random Forests. *Machine Learning* 45, 5–32.	1	142	868	279
Lundberg, S.M., Lee, S.-I. 2017. A Unified Approach to Interpreting Model Predictions. *Advances in Neural Information Processing Systems* 30, arXiv:1705.07874.	4	129	593	168
Friedman, J.H. 2001. Greedy Function Approximation: A Gradient Boosting Machine. *Annals of Statistics* 29(5), 1189–1232.	1	138	571	129
Cortes, C., Vapnik, V. 1995. Support-Vector Networks. *Machine Learning* 20, 273–297.	1	117	512	133
Yap, C.W. 2011. PaDEL-descriptor: An open source software to calculate molecular descriptors and fingerprints. *Journal of Computational Chemistry* 32(7), 1466–1474.	2	103	482	108
Rogers, D., Hahn, M. 2010. Extended-Connectivity Fingerprints. *Journal of Chemical Information and Modeling* 50(5), 742–754.	2	103	461	93
Lundberg, S.M., Erion, G., Chen, H., DeGrave, A., Prutkin, J.M. et al. 2020. From Local Explanations to Global Understanding with Explainable AI for Trees. *Nature Machine Intelligence* 2(1), 56–67.	4	98	356	86
Zhong, S., Zhang, K., Bagheri, M., Burken, J.G., Gu, A. et al. 2021. Machine Learning: New Ideas and Tools in Environmental Science and Engineering. *Environmental Science and Technology* 55(19), 12741–12754.	5	85	286	81
Moriwaki, H., Tian, Y.-S., Kawashita, N., Takagi, T. 2018. Mordred: a molecular descriptor calculator. *Journal of Cheminformatics* 10, 4.	2	89	277	54

**Table 5:** Keyword clusters in ML and environmental chemical research, based on co-occurrence analysis and leading term identification using TF-IDF.

Cluster	Keywords	Count	Leading Keyword (TF-IDF)
1	Artificial Intelligence; Artificial Neural Networks; Big Data; Classification; Climate Change; Deep Learning; Machine Learning; Microplastics; Neural Networks; Precision Agriculture; Spectroscopy; Sustainability	12	Machine Learning
2	Air Pollution; Machine Learning Models; Ozone; Particulate Matter; PM2.5; Prediction; Random Forest; SHAP; Source Apportionment	9	Random Forest
3	Artificial Neural Network; Feature Selection; Genetic Algorithm; Neural Network; Predictive Modeling; Principal Component Analysis; Support Vector Machine; Toxicity Prediction	8	Artificial Neural Network
4	Adsorption; Biochar; Heavy Metals; Modeling; Wastewater Treatment	5	Adsorption
5	Digital Soil Mapping; Ensemble Learning; Remote Sensing; Wastewater; Water Quality	5	Water Quality
6	Cheminformatics; Data Mining; QSAR; QSPR	4	QSAR
7	Computational Toxicology; Risk Assessment; Toxicity; XGBoost	4	Risk Assessment
8	Groundwater; Molecular Docking; PFAS	3	PFAS

## Data Availability

Data will be made available on request.

## Supplementary Materials

The following supporting information can be downloaded at: https://www.mdpi.com/article/10.3390/toxics13100817/s1, Figure S1. International country co-authorship density map for research on ML applications in environmental chemical research. Nodes represent countries meeting the inclusion threshold of ≥10 publications, node size scales with publication output, and color intensity reflects local co-authorship density (total link strength). Layout and clusters were generated in VOSviewer using association-strength normalization. Figure S2. Institutional co-authorship density map for research on ML applications in environmental chemical research. Nodes denote institutions meeting the inclusion threshold of ≥10 publications, node size scales with publication output, and color intensity reflects local co-authorship density (total link strength). Layout and clusters were generated in VOSviewer using association-strength normalization. Figure S3. Source (journal) co-citation density map for research on ML applications in environmental chemical research. Nodes denote journals meeting the inclusion threshold of ≥100 citations, node size scales with citation frequency, and color intensity reflects local co-citation density (total link strength). Layout and clusters were generated in VOSviewer using association-strength normalization. Figure S4. Cited reference co-citation density map for research on ML applications in environmental chemical research. Nodes denote references meeting the inclusion threshold of ≥20 citations (shown as first author, year, source), node size scales with citation frequency, and color intensity reflects local co-citation density (total link strength). Layout and clustering were generated in VOSviewer using association-strength normalization.
