# Supplementary material for: Mapping the Rise in Machine Learning in Environmental Chemical Research: A Bibliometric Analysis
Source: Toxics. 2025 Sep 26;13(10):817. doi: 10.3390/toxics13100817 (PMC12567794; doi:10.3390/toxics13100817)
Supplement: Supplementary file 1 [file toxics-13-00817-s001.zip › toxics-3821623-supplementary.pdf]

## SUPPLEMENTARY MATERIAL

# Mapping the Rise in Machine Learning in Environmental Chemical Research: A Bibliometric Analysis

**Bojana Stanic and Nebojsa Andric** *

Department of Biology and Ecology, Faculty of Sciences, University of Novi Sad, Trg Dositeja Obradovica 2, 21000 Novi Sad, Serbia; bojana.stanic@dbe.uns.ac.rs

* Correspondence: nebojsa.andric@dbe.uns.ac.rs; Tel.: +381-21-485-2675; Fax: +381-21-450-620

## SUPPLEMENTARY FIGURES

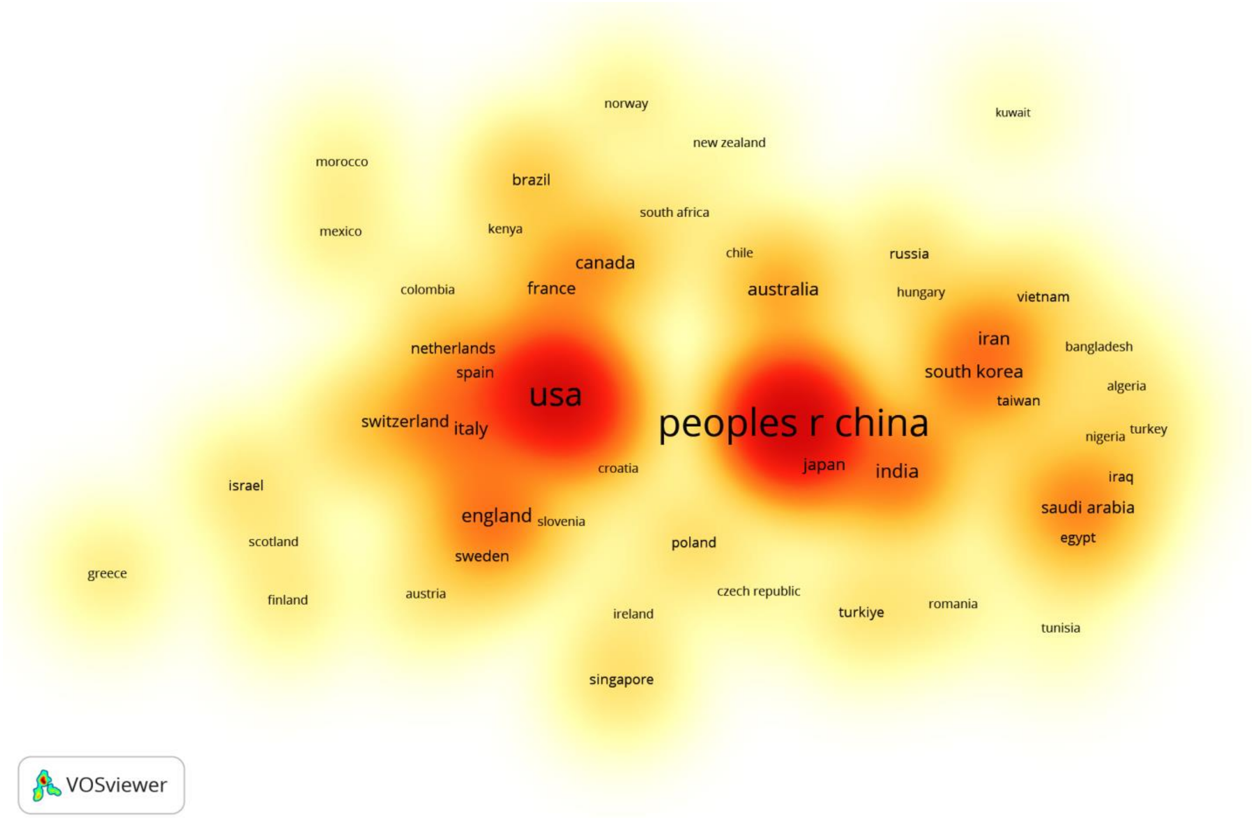


**Figure S1.** International country co-authorship density map for research on ML applications in environmental chemical research. Nodes represent countries meeting the inclusion threshold of ≥10 publications, node size scales with publication output, and color intensity reflects local co-

authorship density (total link strength). Layout and clusters were generated in VOSviewer using association-strength normalization.

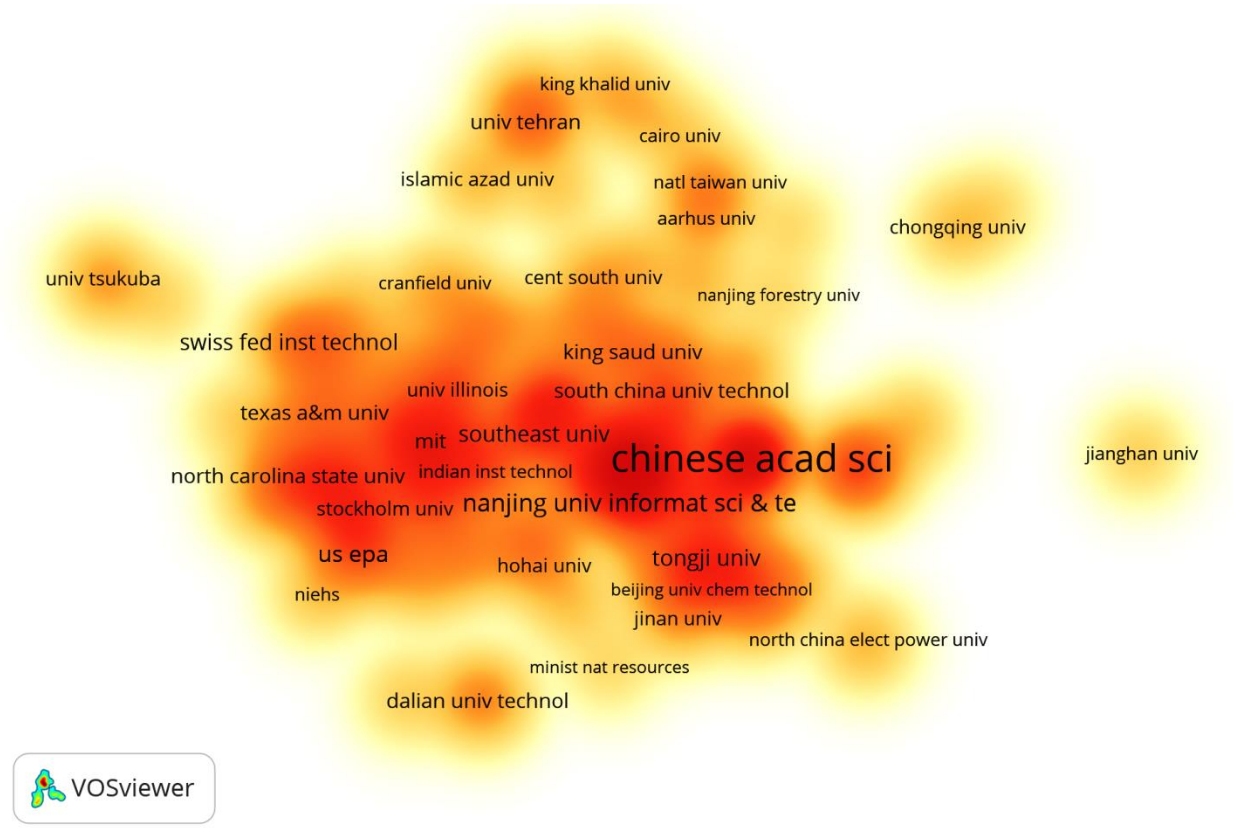


**Figure S2.** Institutional co-authorship density map for research on ML applications in environmental chemical research. Nodes denote institutions meeting the inclusion threshold of ≥10 publications, node size scales with publication output, and color intensity reflects local co-authorship density (total link strength). Layout and clusters were generated in VOSviewer using association-strength normalization.

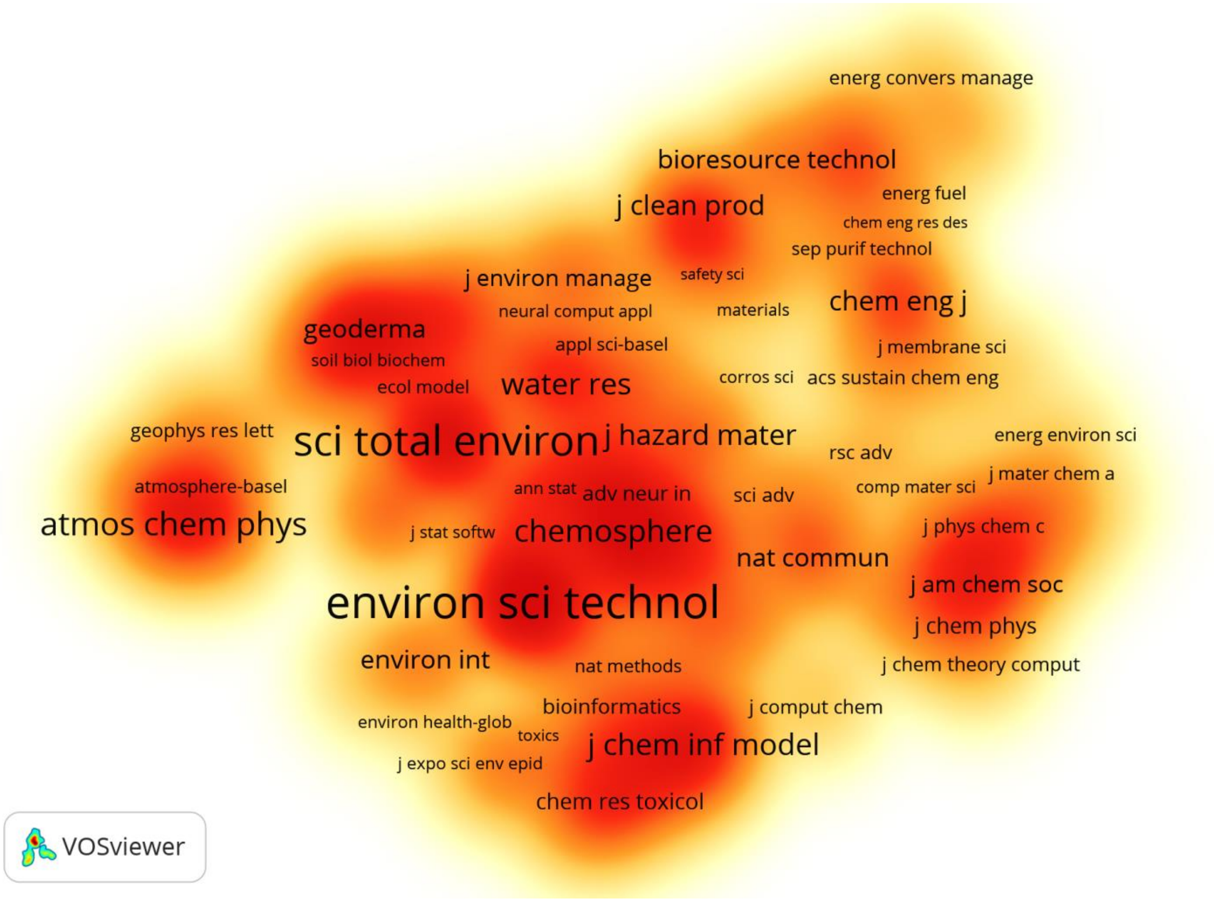


**Figure S3.** Source (journal) co-citation density map for research on ML applications in environmental chemical research. Nodes denote journals meeting the inclusion threshold of ≥100 citations, node size scales with citation frequency, and color intensity reflects local co-citation density (total link strength). Layout and clusters were generated in VOSviewer using association-strength normalization.

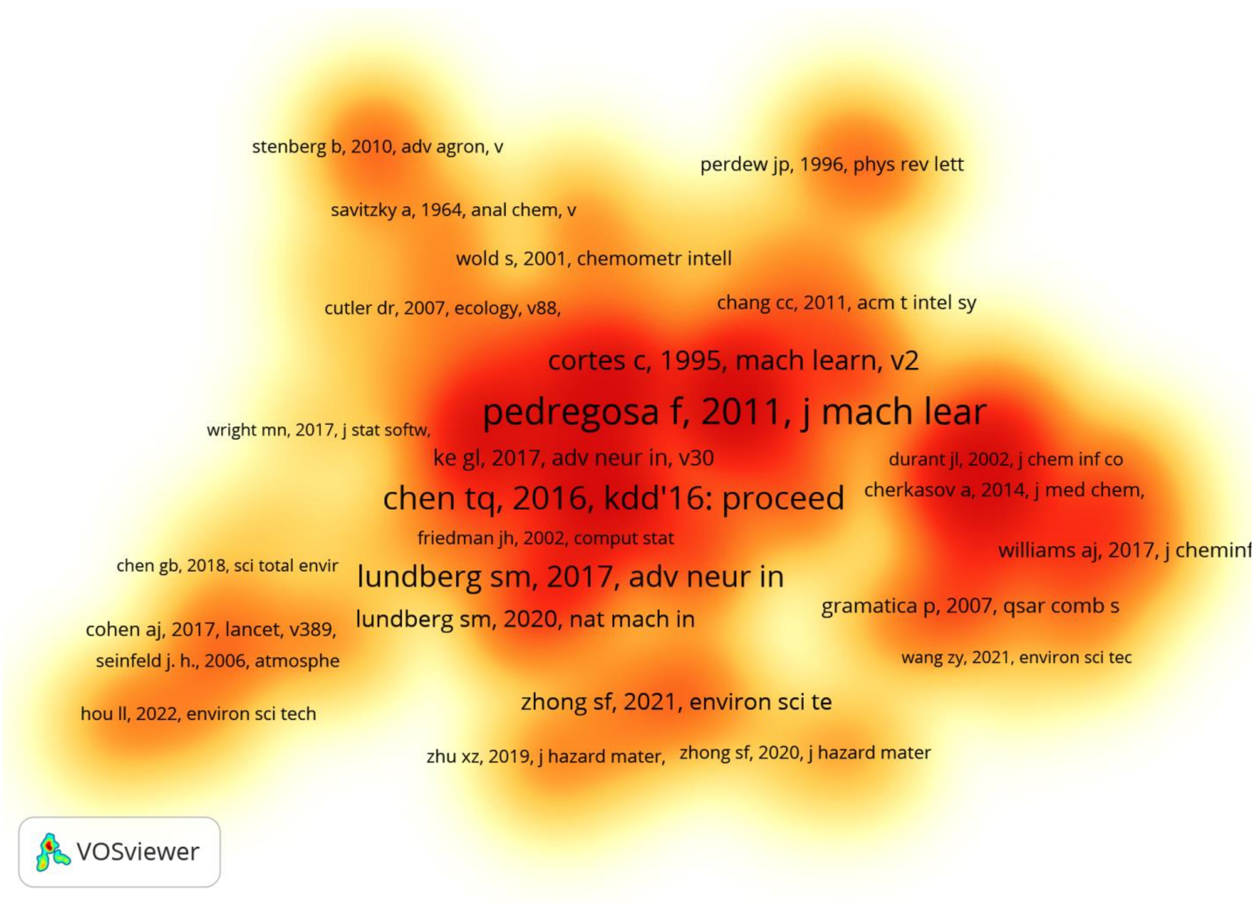


**Figure S4.** Cited reference co-citation density map for research on ML applications in environmental chemical research. Nodes denote references meeting the inclusion threshold of ≥20 citations (shown as first author, year, source), node size scales with citation frequency, and color intensity reflects local co-citation density (total link strength). Layout and clustering were generated in VOSviewer using association-strength normalization.
